# Mental disorders into adulthood among adolescents placed in residential care: A prospective 10-year follow-up study

**DOI:** 10.1192/j.eurpsy.2022.30

**Published:** 2022-06-22

**Authors:** Süheyla Seker, Cyril Boonmann, Delfine d’Huart, David Bürgin, Klaus Schmeck, Nils Jenkel, Martin Steppan, Alexander Grob, Hilma Forsman, Jörg M. Fegert, Marc Schmid

**Affiliations:** 1 Department of Child and Adolescent Psychiatry Research, University Psychiatric Clinics, University of Basel, Basel, Switzerland; 2 Department for Child and Adolescent Psychiatry/Psychotherapy, University of Ulm, Ulm, Germany; 3 Division of Developmental and Personality Psychology, University of Basel, Basel, Switzerland; 4 Department of Social Work, Stockholm University, Stockholm, Sweden

**Keywords:** Developmental psychopathology, longitudinal study, mental disorders, residential care

## Abstract

**Background:**

Child welfare and juvenile justice placed youths show high levels of psychosocial burden and high rates of mental disorders. It remains unclear how mental disorders develop into adulthood in these populations. The aim was to present the rates of mental disorders in adolescence and adulthood in child welfare and juvenile justice samples and to examine their mental health trajectories from adolescence into adulthood.

**Methods:**

Seventy adolescents in shared residential care, placed by child welfare (*n* = 52, mean age = 15 years) or juvenile justice (*n* = 18, mean age = 17 years) authorities, were followed up into adulthood (child welfare: mean age = 25 years; juvenile justice: mean age = 27 years). Mental disorders were assessed based on the International Classification of Diseases 10th Revision diagnoses at baseline and at follow-up. Epidemiological information on mental disorders was presented for each group. Bivariate correlations and structural equation modeling for the relationship of mental disorders were performed.

**Results:**

In the total sample, prevalence rates of 73% and 86% for any mental disorder were found in adolescence (child welfare: 70%; juvenile justice: 83%) and adulthood (child welfare: 83%; juvenile justice: 94%) respectively. General psychopathology was found to be stable from adolescence into adulthood in both samples.

**Conclusions:**

Our findings showed high prevalence rates and a high stability of general psychopathology into adulthood among child welfare and juvenile justice adolescents in Swiss residential care. Therefore, continuity of mental health care and well-prepared transitions into adulthood for such individuals is highly warranted.

## Introduction

Children and adolescents in the child welfare system experience high levels of cumulative familial and psychosocial burden such as maltreatment, mental health issues, delinquent behavior, and low socioeconomic status [1–3]. Youths in the juvenile justice system are marked by similarly elevated rates of family dysfunction, psychopathology, and other psychosocial burdens [4, 5]. These burdens can elevate the risk of chronic mental health issues and other psychosocial difficulties into adulthood [6, 7]. A substantial number of youths involved with the child welfare system cross over to the juvenile justice system or vice versa, meaning that these youths can be dually involved in both systems [8, 9]. In Switzerland, for example, the juvenile justice system is an offender-oriented criminal law system with an explicit focus on rehabilitation [10], meaning that the protection of juvenile justice placed adolescents’ development takes priority over punitive aspects. In contrast to many other countries, child welfare youths and juvenile delinquents can thus be resident in the same facilities in Switzerland. To date, it remains unclear to what extent the mental disorder status of child welfare and juvenile justice placed youths in residential care develops after their exit from care in adulthood.

In a meta-analysis of children and adolescents in the child welfare system (including residential and foster care settings), a pooled prevalence rate of 49% was found for at least one present mental disorder [11]—this rate was higher compared with peers in the general population [12]. In juvenile justice samples, a prevalence rate of 70% for any mental disorder (including present and lifetime diagnoses) has been found (see the literature review of [13]). Previous literature reviews and meta-analyses examining the development of youths’ mental health problems in out-of-home care have found that general behavioral problems were stable during foster care [14, 15]; no evidence was found that growing up in care is generally ameliorating or detrimental for children who enter care [16]. In jurisdictions where children predominantly enter care following severe and persistent maltreatment, a child’s age at entry into care strongly predicts their subsequent mental health issues and younger age at entry is protective [17]. Furthermore, longitudinal studies have identified that youths incur further deterioration in their mental health following placement disruptions (e.g., [18, 19]) or attachment insecurity (e.g., [20]).

Monitoring the mental health status of youths in child welfare or juvenile justice systems is crucial, as disorders that manifest in childhood and adolescence, especially for individuals with numerous risk factors, carry a high risk of persisting into adulthood and severely influencing long-term functionality [21–26]. In particular, externalizing disorders in adolescence have been found to be persistent and substantially affect adjustment in early adulthood in the general population and in high-risk samples [27, 28]. Especially young adulthood can be a vulnerable period where the risk of emergence and stability of mental disorders is particularly high [29, 30]. It is acknowledged that out-of-home placed youths often face various challenges related to mental disorders in their transition from in-care to out-of-care, often when they turn 18 and have to leave out-of-home care in the transition to young adulthood (e.g., homelessness, adult mental health issues, or unemployment; [6, 31–33]). Previous studies have revealed that older youths in foster care show disproportionally high rates of lifetime and past-year psychiatric disorders (e.g., [34]), and adults with a history of child welfare out-of-home care showed a prevalence rate of 30% for any mental disorder [35]. Similarly, prospective and retrospective studies have emphasized that adolescents who were placed within the juvenile justice system suffer from multiple mental health problems in young adulthood [36, 37]; adults with a juvenile justice placement history showed an even higher rate of 45% for any mental disorder in adulthood [35]. The elevated rates of childhood adversities [38] and the challenging transition from foster or residential care to an independent adult life have been associated with poor mental health for these emerging adults [39, 40]; the risk of various forms of disadvantage, such as mental health issues, can persist even into midlife (e.g., [41]).

From a developmental perspective, behavioral genetic studies found that longitudinal stability in psychological traits among representative samples of twins is predominantly explained by shared genetic influences, whilst changes in the same measures across time are instead attributed to environmental factors [42]. A recent Finnish co-sibling cohort study of 885,662 children and adolescents identified a wide range of placement characteristics, including institutional placements and placement instability, that were associated with several measures of psychiatric disorders, even following adjustments for unmeasured familial confounding [43]. Similarly, parental mental illness, which is common among out-of-home placed individuals, is a risk factor for child mental disorders, suicidal ideation and attempts [44], and increased attachment insecurity [45]. As Axis-I and Axis-II disorders are comparably stable over time with regard to the underlying diagnostic groups, the general dimension *p*-factor of psychopathology, which is incidentally also considerably heritable, should therefore be expanded [46, 47].

To the best of our knowledge, there is a lack of longitudinal studies of mental disorders assessed with standardized clinical diagnoses from childhood and adolescence into adulthood among child welfare and juvenile justice samples. Further, the development of child welfare and juvenile justice placed youths in Swiss residential care into adulthood (i.e., after they leave care) remains unclear. Thus, the aim of this prospective 10-year follow-up study was twofold: (a) to examine the prevalence rates of mental disorders among child welfare and juvenile justice placed adolescents in residential care and in young adulthood, and (b) to study mental health trajectories from adolescence into young adulthood in these samples.

## Methods

### Study design and procedure

The data used in this study originate from the longitudinal “Swiss Study for Clarification and Goal-Attainment in Youth Welfare and Juvenile Justice Institutions” (German: Modellversuch Abklärung und Zielerreichung in stationären Massnahmen [MAZ.]) [48] and the follow-up study “Youth Welfare Trajectories: Learning From Experience” (German: Jugendhilfeverläufe: Aus Erfahrung lernen [JAEL]). Overall, 592 children, adolescents, and young adults aged 6−26 years participated in the MAZ. study and 231 participants were included in the follow-up JAEL study.

The MAZ. study team contacted every child welfare and juvenile justice residential care institution in Switzerland accredited by the Federal Office of Justice, and a total of 64 institutions agreed to take part, yielding a representative sample of the different institution types (e.g., large versus small institutions, institutions with or without internal schools, and internal versus external access to treatment programs) as well as the heterogenous groups of youths who reside in them [48]. Subsequently, all children, adolescents, and young adults (and their legal representatives) meeting the eligibility criteria (i.e., living in one of the aforementioned 64 institutions for at least 1 month prior to the study, and having sufficient cognitive capacity and knowledge of the German, French, or Italian language to answer the questionnaires and interviews) were contacted, as well as their caseworkers. Informed consent for participation in the study was obtained from the children and adolescents and their legal representatives. Trained psychologists from the study team conducted well-established, semi-structured clinical interviews and multiple psychometric questionnaire surveys with the participants.

The aim of the follow-up JAEL study (mean follow-up = 9.7 years) was to reexamine MAZ. participants using Web-based psychometric questionnaires, as well as face-to-face (semi-structured, clinical) interviews, about mental health problems and disorders, personality, delinquency, quality of life, and retrospective experiences in residential out-of-home care. The procedures of MAZ. and JAEL were approved by the Ethics Committee on Research Involving Humans at the University of Basel. The present study was reported and conducted following the “Strengthening the Reporting of Observational Studies in Epidemiology” (STROBE) statement [49].

### Participants

First, adolescents had to be placed by either the child welfare (civil law) or the juvenile justice (criminal law) authority for inclusion in the present study. Second, the upper age limit in the MAZ. study was set at 18 years of age, which is usually when child welfare placements end. This resulted in 343 eligible MAZ. participants for the present study (see Supplementary Figure S1). Participants (42.7%) with missing data for our included variables were further excluded (missing values varied from 26.2 to 72.0% across the measures). Hence, the total available sample was reduced to a study sample of 70 participants (52 child welfare and 18 juvenile justice youths). Participants at the time of the MAZ. assessment were on average 15.65 years old (standard deviation = 1.48, age range = 12–18 years). The mean age of the sample was 25.89 years (standard deviation = 1.66 years, age range = 21–29 years) at the time of the JAEL study, with 25 (36.0%) female participants and 60 (85.0%) participants with Swiss citizenship.

The sample attrition analysis revealed that participants in the present study (complete cases: *n* = 70) did not differ significantly from those who did not participate in the follow-up JAEL study (*n* = 273) in terms of sociodemographic characteristics and adolescent mental disorders (see Supplementary Table S1). Thus, the analyses in the present study were based on the Missing at Random assumption and conducted with the complete cases data set.

### Measurements

#### Sociodemographic characteristics

In the MAZ. study, an anamnestic computer-based questionnaire, created by the MAZ. study team, assessed sociodemographic information (age, gender, Swiss citizenship [1 = yes, 0 = no], age at first entry into care, number of placements, and duration of care [total time spent in out-of-home care]) and was filled out by the adolescents’ caregivers (i.e., caseworkers).

#### Mental disorders

At baseline, childhood and adolescent mental disorders were assessed with the Kiddie Schedule for Affective Disorders and Schizophrenia—Present and Lifetime Version (K-SADS-PL; [50]). K-SADS-PL is a standardized, semi-structured clinical interview framework for the assessment of mental disorders in children and adolescents aged 6–18 years following the fourth edition of the Diagnostic and Statistical Manual of Mental Disorders (DSM-IV; [51]). The individual responses are rated on a 4-point Likert scale (0 = no information available, 1 = not present, 2 = subthreshold level, 3 = threshold level). The psychometric properties of K-SADS-PL have been found to be good [52]. For the present study, only present diagnoses assessed with K-SADS-PL were included, and diagnoses were classified according to the International Classification of Diseases 10th Revision (ICD-10) system.

At follow-up, adult mental disorders were assessed with the Structured Clinical Interview for DSM-5 Disorders—Clinician Version (SCID-5-CV; [53]). SCID-5-CV is a semi-structured clinical interview based on the adult disorder dimensions from DSM-5 and is implemented with adults (i.e., > 18 years). The items and diagnoses were rated with dichotomous response options consisting of 1 = present and 0 = not present. For this study, present diagnoses assessed with SCID-5-CV were included and diagnoses were classified according to the ICD-10 system. For the assessment of Axis-I disorders in childhood and adulthood, longitudinal studies usually administer K-SADS to participants < 18 years and SCID-5 to participants > 18 years and have shown good validity for both clinical interviews [54].

The Structured Clinical Interview for DSM-IV-TR Axis II Personality Disorders (SCID-II; [55]) was used to assess personality disorders in adolescence (i.e., baseline) and in adulthood (i.e., follow-up). The SCID-II interview consists of 134 items, which are rated on a 3-point Likert scale (1 = absent, 2 = subthreshold, 3 = threshold). In SCID-II, categorical diagnoses are obtained according to the DSM-IV diagnostic threshold for individual personality disorders. The inter-rater reliability for categorical diagnoses with SCID-II varies from 0.48 to 0.98 (Cohen’s κ), and internal consistency ranges from 0.71 to 0.94 [80].

For grouping the adolescent mental disorders and the adult mental disorders, we arranged the ICD-10 F-coded disorders into groups following the valid classification system of disorders in the Hierarchical Taxonomy of Psychopathology (HiTOP) model (see Supplementary Table S2; http://medicine.stonybrookmedicine.edu/HITOP; [56–58]). In contrast to traditional diagnostic systems, the HiTOP model constructs psychopathological syndromes (i.e., sexual problems, eating disorders, fear, distress, mania, substance abuse, antisocial behavior) and their subtypes based on the observed covariation of symptoms, thus reducing heterogeneity, and combines these co-occurring psychopathological syndromes into spectra (i.e., somatoform, internalizing, thought disorder, disinhibited externalizing, antagonistic externalizing, detachment; [57]).

#### Mental disorder trajectory groups

Based on the dichotomous “any mental disorder” categorization, four mental disorder trajectory groups were built: those with a mental disorder (not necessarily the same one) at baseline and follow-up, those with a mental disorder at follow-up but not at baseline, those without a disorder at baseline and follow-up, and those with a mental disorder that had improved at follow-up.

### Statistical analyses

First, absolute and relative frequencies of the mental disorder spectra, as well as sociodemographic characteristics, were drawn up for the total sample and for the child welfare and juvenile justice samples. Group comparisons for child welfare and juvenile justice adolescents and sociodemographic variables were made using the *t*-test or the χ^2^-test. To compare groups with different psychopathological trajectories, one-factor analysis of variance (ANOVA) was applied using groups of mental disorders as the between-subject factor.

Second, tetrachoric correlations for adolescent and adult mental disorders were calculated for the total, child welfare, and juvenile justice samples. The tetrachoric correlation coefficient measures the relationship between two dichotomous baseline and follow-up variables with the assumption of bivariate normality [59]. The *p*-factor of general psychopathology is normally distributed within the general population [60], and tetrachoric correlations account for a change from a dimensional to a categorical paradigm of mental disorders, which is what the diagnostic interviews in our study assessed. We used a Bonferroni correction to avoid multiple testing bias for the tetrachoric correlations. In multiple hypothesis testing, the Bonferroni correction is a conservative method for probability thresholding to adjust for an alpha (α) error (rates of false positives; [61]). Due to a high correlation of the “externalizing antagonistic” and “externalizing disinhibited” disorder spectra in adolescence (child welfare: *r_tet_* = 0.99; juvenile justice: *r_tet_* = 0.92) and in adulthood (child welfare: *r_tet_* = 0.96; juvenile justice: *r_tet_* = 0.92), both spectra were grouped together in a single category labeled “externalizing disorders” spectrum.

Third, to test the latent association between adolescent mental disorders and adult mental disorders in young adulthood, a confirmatory factor analysis (CFA) structured according to the HiTOP model was performed [62]. Factor loadings, covariances of the latent intercept, and the slope were freely estimated. The model fit was evaluated with the χ^2^-test, assuming the following null hypothesis: the model-implied variance-covariance matrix (i.e., HiTOP model) is equivalent to the empirical variance-covariance matrix. A nonsignificant *p*-value (>0.05) for the χ^2^-test is evaluated as a good model fit and, thus, indicates that the null hypothesis is accepted. Model fit indices such as root mean square error of approximation (RMSEA), comparative fit index (CFI), and Tucker–Lewis index (TLI) were evaluated. RMSEA values < 0.05 and CFI and TLI values > 0.95 were considered to indicate a good model fit [63]. Considering these different fit indices provides a general summary of the model fit and aids evaluation of the best model that fits the observed data.

Next, a multigroup CFA was conducted to examine measurement invariance of the HiTOP model across the child welfare and juvenile justice samples. Multigroup measurement invariance analysis was investigated by estimating competing models with different levels of constraint: configural, weak factorial (metric), and strong factorial (scalar) [64]. Measurement invariance was tested by comparing the configural, weak factorial, and strong factorial models with an ANOVA test that is based on a χ^2^ difference test for the models [65]. In CFA, strong factorial invariance is preferable as it indicates that the measurement model (i.e., HiTOP model) has the same structure across groups, suggesting the validity of the HiTOP model and factor loadings in both groups. Additionally, to compare differences between child welfare and juvenile justice samples, group differences in the temporal stability of general psychopathology factor scores (i.e., participants’ position on the latent psychopathology dimension in adolescence and adulthood) from adolescence to adulthood were tested using Fisher’s *z* test.

The 95% confidence interval (CI) was presented for prevalence rates of mental disorder estimates and for parameter estimates in the CFA models. All models were calculated with a structural equation modeling approach using the “lavaan” package [66] with the statistical software R (version 4.0.2; [67]). For all analyses, *p*-values were two-tailed, and differences were considered significant at *p* < 0.05.

## Results

### Sociodemographic characteristics

Sociodemographic characteristics for the total sample, and by adjudicating court (i.e., child welfare or juvenile justice), are presented in Table 1. Juvenile justice placed youths were older (*t*(43.63) = −4.02, *p* < 0.001), were older at first entry into care (*t*(19.86) = −4.73, *p* < 0.001), and spent less time in care (*t*(57.35) = 3.26, *p* < 0.01) compared to child welfare placed youths.

**Table 1.** Group differences in baseline sociodemographic characteristics.

Variable	Total (*N* = 70)	Child welfare (*n* = 52)	Juvenile justice (*n* = 18)	Test statistic
Age in years (*M,* SD)	15.65 (1.48)	15.33 (1.48)	16.59 (1.01)	*t*(43.63) = −4.02***
Number of placements (*M,* SD)	3.63 (3.02)	3.62 (3.24)	3.67 (2.35)	*t*(40.85) = −0.07, n.s.
Age at first entry into care in years (*M,* SD)	10.23 (4.68)	8.61 (4.45)	14.54 (1.42)	*t*(19.86) = −4.73***
Duration of care in years (*M,* SD)	6.28 (4.75)	7.08 (5.08)	4.00 (2.63)	*t*(57.35) = 3.26**
Gender (% [*n*])				χ^2^(1) = 0.28, n.s.
Female	35.7 (25)	38.5 (20)	27.8 (5)	
Male	64.3 (45)	61.5 (32)	72.22 (13)	
Swiss citizenship (% [*n*])	85.7 (60)	86.5 (45)	83.3 (15)	χ^2^(1) = 0.00, n.s.

Abbreviations: M, mean; n.s., not significant; SD, standard deviation.

** *p* < 0.01;

*** *p* < 0.001.

### Prevalence rates of mental disorders

In adolescence, 72.9% (95% CI = 62.4, 83.2) of the total sample showed any mental disorder (see Table 2), and in adulthood 85.7% (95% CI = 77.5, 93.9) of the participants had any mental disorder (see Table 3). Externalizing disorders (*n* = 45, 64.3% [95% CI = 53.1, 75.5]) and internalizing disorders (*n* = 20, 28.6% [95% CI = 18.0, 39.2]) were the most prevalent disorder spectra in adolescence, which was in line with the pattern in adulthood (externalizing disorders: *n* = 58, 82.9% [95% CI = 74.0, 91.7]; internalizing disorders: *n* = 26, 37.1% [95% CI = 25.8, 48.7]). For specific disorder groups, the highest prevalence rate in adolescence was found for antisocial behavior disorders (*n =* 41, 58.6% [95% CI = 47.0, 70.1]), followed by distress disorders (*n* = 16, 22.9% [95% CI = 13.0, 32.7]), substance abuse (*n* = 15, 21.4% [95% CI = 11.2, 31.0]), and fear disorders (*n* = 9, 12.9% [95% CI = 5.0, 20.7]). A similar pattern was observed in adulthood (antisocial behavior disorders: *n* = 53, 75.7% [95% CI = 65.7, 85.8]; substance abuse: *n* = 29, 41.2% [95% CI = 29.9, 53.0]; distress disorders: *n* = 23, 32.9% [95% CI = 21.9, 43.9]; fear disorders: *n* = 11, 15.7% [95% CI = 7.1, 24.2]). In adolescence, juvenile justice placed youths showed substance abuse more often compared to child welfare placed adolescents (χ^2^(1) = 3.10, *p* < 0.05). In adulthood, juvenile justice placed participants showed antisocial behavior disorders more often compared to child welfare placed participants (χ^2^(1) = 3.35, *p* < 0.05).

**Table 2.** Prevalence rates and univariate group differences of adolescent mental disorders in child welfare and juvenile justice samples (% [*n*]).

	Adolescent mental disorders						
Disorder group	Total (*n* = 70)	95% CI	Child welfare (*n* = 52)	95% CI	Juvenile justice (*n* = 18)	95% CI	Test statistic
Fear disorders	12.9 (9)	5.0, 20.7	11.5 (6)	2.9, 20.2	16.7 (3)	0, 33.9	χ^2^(1) = 0.02, n.s.
Distress disorders	22.9 (16)	13.0, 32.7	21.2 (11)	10.1, 32.3	27.8 (5)	7.1, 48.5	χ^2^(1) = 0.06, n.s.
Mania disorders	1.0 (1)	0, 4.2	1.0 (1)	0, 5.7	0 (0)	N/A	χ^2^(1) = 0.00, n.s.
Eating disorders	0 (0)	N/A	0 (0)	N/A	0 (0)	N/A	N/A
Substance abuse	21.4 (15)	11.2, 31.0	15.4 (8)	5.6, 25.2	38.9 (7)	16.4, 61.4	χ^2^(1) = 3.10*
Antisocial behavior disorders	58.6 (41)	47.0, 70.1	55.8 (29)	42.3, 69.3	66.7 (12)	44.9, 88.4	χ^2^(1) = 0.28, n.s.
Disorder spectrum							
Thought disorders	4.3 (3)	0, 9.0	1.9 (1)	0, 5.7	11.1 (2)	0, 25.6	χ^2^(1) = 0.97, n.s.
Internalizing disorders	28.6 (20)	18.0, 39.2	26.9 (14)	14.9, 39.0	33.3 (6)	11.6, 55.1	χ^2^(1) = 0.93, n.s.
Externalizing disorders	64.3 (45)	53.1, 75.5	59.6 (31)	46.3, 73.0	77.8 (14)	58.6, 97.0	χ^2^(1) = 1.21, n.s.
Detachment disorders	5.7 (4)	0.3, 11.2	3.9 (2)	0, 9.1	11.1 (2)	0, 25.6	χ^2^(1) = 0.31, n.s.
Any mental disorder	72.9 (51)	62.4, 83.2	69.2 (36)	62.4, 83.3	83.3 (15)	62.4, 83.3	χ^2^(1) = 2.36, n.s.

Abbreviations: CI, confidence interval; N/A, not applicable; n.s., not significant.

* *p* < 0.05.

**Table 3.** Prevalence rates and univariate group differences of adult mental disorders in child welfare and juvenile justice samples (% [n]).

	Adult mental disorders						
Disorder group	Total (*n* = 70*)*	95% CI	Child welfare (*n* = 52)	95% CI	Juvenile justice (*n* = 18)	95% CI	Test statistic
Fear disorders	15.7 (11)	7.1, 24.2	17.3 (9)	7.0, 27.6	11.1 (2)	0, 25.6	χ^2^(1) = 0.06, n.s.
Distress disorders	32.9 (23)	21.9, 43.9	30.8 (16)	18.2, 43.3	38.9 (7)	16.4, 61.4	χ^2^(1) = 0.12, n.s.
Mania disorders	1.0 (1)	0, 4.2	1.0 (1)	0, 5.7	0 (0)	N/A	χ^2^(1) = 0.00, n.s.
Eating disorders	N/A	N/A	N/A	N/A	N/A	N/A	N/A
Substance abuse	41.2 (29)	29.9, 53.0	42.3 (22)	28.9, 55.7	38.9 (7)	16.4, 61.4	χ^2^(1) = 0.00, n.s.
Antisocial behavior disorders	75.7 (53)	65.7, 85.8	69.2 (36)	42.3, 69.3	94.4 (17)	83.9, 1.0	χ^2^(1) = 3.35*
Disorder spectrum							
Thought disorders	10.0 (7)	3.0, 17.0	5.8 (3)	0, 12.1	22.2 (4)	3.0, 41.4	χ^2^(1) = 2.40, n.s.
Internalizing disorders	37.1 (26)	25.8, 48.7	36.5 (19)	23.5, 49.6	38.9 (7)	11.6, 55.1	χ^2^(1) = 0.00, n.s.
Externalizing disorders	82.9 (58)	74.0, 91.7	78.9 (41)	67.7, 89.9	94.4 (17)	83.9, 100.0	χ^2^(1) = 1.32, n.s.
Detachment disorders	10.0 (7)	3.0, 17.0	11.5 (6)	2.9, 20.2	5.6 (1)	0, 16.1	χ^2^(1) = 0.08, n.s.
Any mental disorder	85.7 (60)	77.5, 93.9	82.7 (43)	77.5, 93.9	94.4 (17)	62.4, 83.3	χ^2^(1) = 0.70, n.s.

*Note:* Eating disorders are not included as a diagnosis in the SCID-5-CV and were thus not assessed in adulthood.

Abbreviations: CI, confidence interval; N/A, not applicable; n.s., not significant; SCID-5-CV, structured clinical interview for DSM-5 disorders-clinician version.

* *p* < 0.05.

### Trajectories of adolescent and adult mental disorders

First, the trajectory group with any mental disorder at baseline and follow-up was the largest group (*n* = 45, 64.3%) in the total sample, followed by the trajectory group with a mental disorder at follow-up but not at baseline (*n* = 15, 21.4%), the group with a mental disorder that had improved through adulthood (*n* = 6, 8.6%), and, finally, the group without a mental disorder at both baseline and follow-up (*n* = 4, 5.7%). The mental disorder trajectory groups did not differ in sociodemographic characteristics and by adjudicating court (see Supplementary Table S3).

Second, the bivariate correlation matrix of adolescent and adult mental disorders in the total sample is shown in the Supplementary Figure S2, and the bivariate correlation matrices of adolescent and adult mental disorders by adjudicating court are presented in the Supplementary Figures S3 and S4.

**Figure 1.**
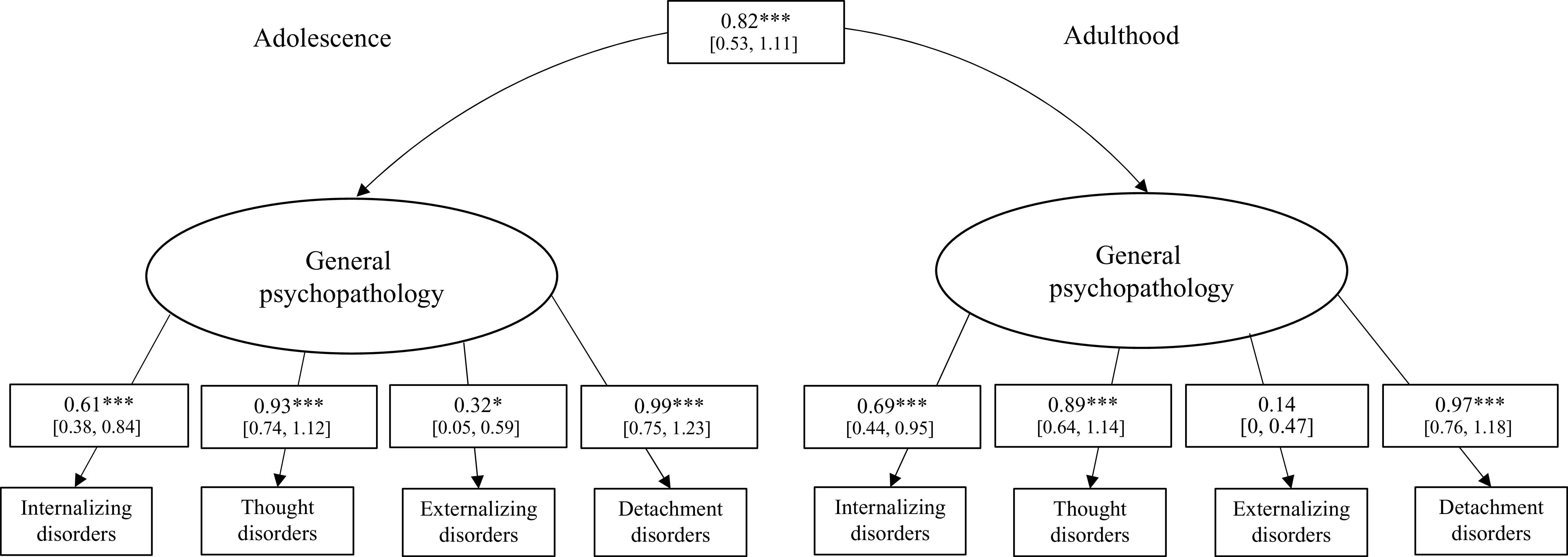
Two-factor confirmatory factor analysis of adolescent and adult mental disorders in the total sample (*n* = 70). CI, confidence interval. The 95% confidence interval for factor loadings is presented in brackets. *p < 0.05, ^***^p < 0.001.

Third, the two-factor CFA showed a nonsignificant χ^2^-test (χ^2^(15) = 13.24, *p* = 0.58), indicating a good model fit. Furthermore, the CFA also provided good fit indices for RMSEA = 0.00, CFI = 1.00, and TLI = 1.03. All factor loadings of the indicator variables (mental disorder groups) were significant for the adolescent general psychopathology latent variable (*p* < 0.05 in all cases, see Figure 1). For the adult general psychopathology latent variable, the internalizing, thought, and detachment disorder indicator variables showed significant factor loadings (*p* < 0.001 in all cases), whereas the factor loading for the externalizing disorders indicator variable was not significant (*p* = 0.39). There was a significant covariance between adolescent and adult general psychopathology (*b* = 0.82, SE = 0.15, *p* < 0.001), indicating temporal stability of general psychopathology.

**Figure 2.**
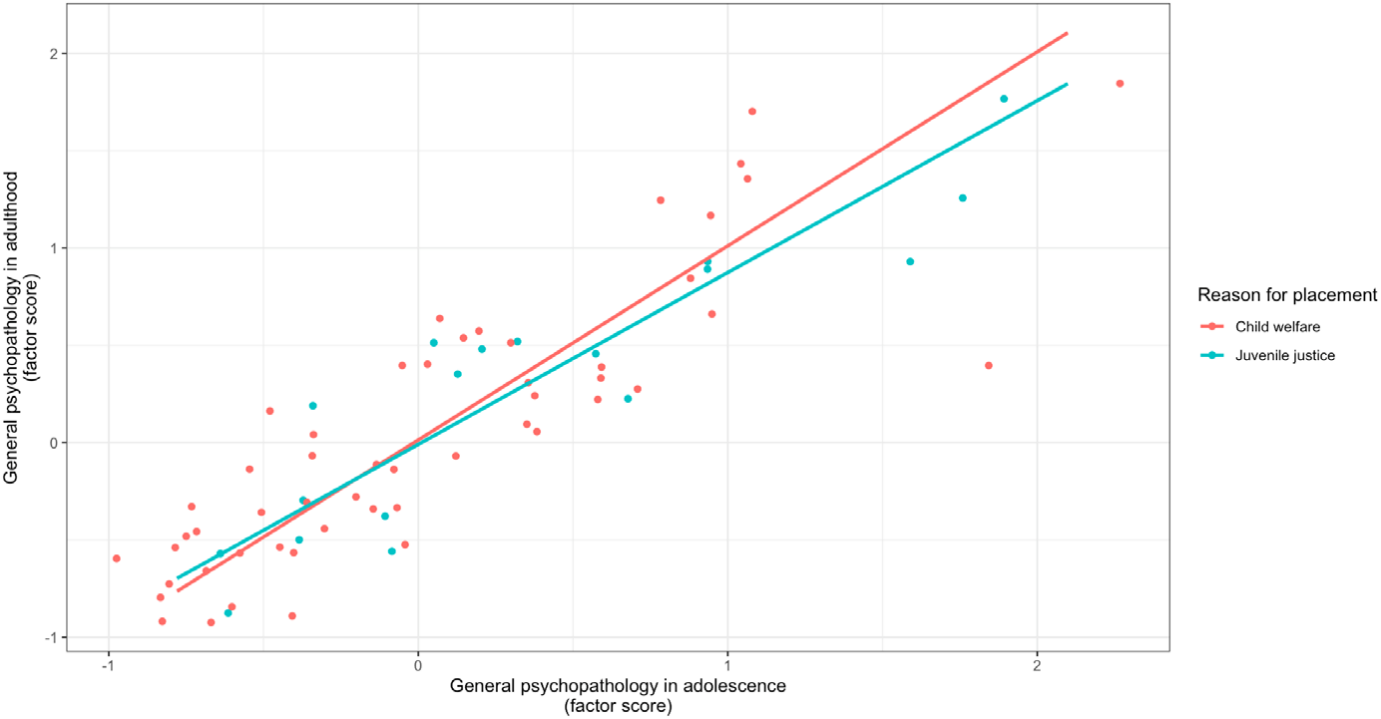
Temporal stability of adolescent and adult general psychopathology in the child welfare (*n* = 52) and juvenile justice sample (*n* = 18). The *x*- and *y*-axes are scaled according to the factor score of adolescent and adult general psychopathology derived from the multigroup confirmatory factor analysis. The factor scores between both groups did not differ significantly between the child welfare and juvenile justice group.

A measurement invariance analysis comparing adolescent and adult mental disorders between the child welfare and juvenile justice groups revealed that the scalar invariance was the best suited model (χ^2^(6) = 4.73). This model outperformed configural invariance and metric invariance in terms of model fit. Thus, the same factorial structure, including similar factor loadings and intercepts, that is, strong measurement invariance, can be assumed, corroborating the validity of the HiTOP model in both groups. Lastly, Fisher’s *z* test for the adolescent general psychopathology and adult general psychopathology factor scores revealed no significant differences between the child welfare and juvenile justice samples (*z* = −0.48, *p* = 0.63), indicating a similarly large temporal stability of general psychopathology in both samples (see Figure 2).

## Discussion

To the best of our knowledge, this is the first longitudinal study to examine the prevalence rates and trajectories into adulthood of mental disorders, assessed with standardized diagnostic systems, in a high-risk group of child welfare and juvenile justice placed adolescents in Swiss residential care.

Almost 73% of adolescents in out-of-home care showed any mental disorder. The rates for any mental disorder were similar in both the child welfare (70%) and the juvenile justice (83%) samples. Our results are consistent with the findings of previous meta-analyses of mental disorders among adolescents in the juvenile justice system [13] and adolescents placed by the child welfare system [11], and these rates are far higher compared to a pooled prevalence rate (13%) for any mental disorder among children and adolescents in the general population [12]. In the present study, the prevalence rate of 86% for any mental disorder among the total sample in adulthood (child welfare: 83%; juvenile justice: 94%) is higher than in a recent meta-analysis of mental disorders in adults formerly placed in out-of-home care by child welfare or juvenile justice authorities [35], and also far higher than a pooled prevalence rate of 18% for any mental disorder in the general adult population [68]. The high prevalence rates found in the present study may be due to the inclusion of both child welfare and juvenile justice youths. It may be that these youths—placed by either civil or criminal law measures—are marked by similarly high (trauma-related) psychopathology, individual behavioral difficulties, and various psychosocial burden.

Within our total sample, around 65% of participants showed persistent general psychopathology. A large temporal stability for general psychopathology from adolescence into adulthood was found, and this pattern holds for both child welfare and juvenile justice samples. Our findings are in line with previous research in a general population indicating that childhood mental disorders are significantly associated with adult mental disorders [69]. The high stability of general psychopathology can be explained by the single dimension of general psychopathology, *p*, which may account for nonspecificity in psychopathology [46]. Adolescence and young adulthood in general are particularly vulnerable periods for the emergence and stability of mental disorders [30], especially for young adults who age out-of-care. They may face various challenges during the transition to an independent adult life (e.g., less social support and mental health issues) and thus might be at a higher risk of mental disorders compared with older adults [70]. In the USA, for example, the federal government passed legislation to fund independent living services to support older youths in care with adequate skills for a successful transition out-of-care, as well as social and economic support [71]. In Switzerland, adolescents usually leave residential care on reaching adulthood (i.e., 18 years of age): these young adults, compared to their peers in the general population, often do not have the opportunity to return to a parental home upon leaving the institution or have few social support options.

### Limitations and implications

First, the mixed sample of child welfare and juvenile justice youths in Swiss residential care institutions has the potential to examine treatment needs in both samples [72]. Nonetheless, our Swiss sample represents a unique sample in that our results are not generalizable to other countries given the different regulations and jurisdictions. The relatively small sample size due to our inclusion and exclusion criteria, as well as the proportion of missing data, prevented us from including further subgroup analyses (e.g., gender or childhood adversities).

Second, the time period of 10 years between the two study points (i.e., MAZ. and JAEL) could mean that other factors between the study points that could not be recorded could have had an influence on mental disorders. The present findings do not allow any conclusions to be drawn about the effects of residential placements per se, especially because effective control groups (e.g., adolescents with childhood adversities but no out-of-home placements) are lacking. Also, the information regarding the use of mental health services during and after care in this Swiss sample is limited. Where treatment options are concerned, mention can be made of a German online training application that was developed for clinical and socio-pedagogical practitioners working with young people in the transition from child and adolescent psychiatry to general adult psychiatry, aiming to convey expertise in transition psychiatry and care delivery systems (see https://www.uniklinik-ulm.de/kinder-und-jugendpsychiatriepsychotherapie/forschung-und-arbeitsgruppen/arbeitsgruppe-wissenstransfer-dissemination-e-learning/protransition/protransition-engl.html).

As adolescence and young adulthood are vulnerable age stages for the development of mental disorders, early identification and tailored intervention planning should be an integral part of a placement and admission process in which the resources and strengths of these adolescents should be adequately assessed [73]—especially by offering continuity in evidence-based psychotherapy and well-prepared transitions [15, 74–76]. For example, primary treatment modalities (e.g., family-based therapies and multicomponent interventions) have been suggested as primary strategies for treating substance-use disorders among adolescents, and a dialectical-behavioral therapy plus milieu approach demonstrated sustained positive treatment outcomes [77, 78]. Furthermore, evidence-based practices have been shown to be relevant to group care and residential treatment settings for children in the child welfare system [74]. In light of our findings regarding the high stability of psychopathology among young people in residential care, practitioners should thus aim to lower the barriers for adolescents and young adults in seeking and accepting support by motivating them to participate in effective and easily accessible evidence-based therapies.

Third, Axis-I mental disorders were assessed with two different instruments (K-SADS-PL and SCID-5-CV) in our study, which leads to a slightly different classification and assessment of disorders in adolescence and adulthood. However, both instruments classify mental disorders according to the ICD-10 diagnostic system, which we included in our study. Although dichotomous diagnoses are used in clinical practice, our sample may nonetheless include some participants with high subthreshold ratings. Questionnaires tend to be more stable than interviews, and dimensional ratings show higher stability compared to categorical ratings [79]. Hence, future studies with samples of children and adolescents in different care settings, comparing mental disorders categorically and dimensionally, are desirable.

## Conclusion

The main finding of the present study is that almost three quarters of adolescents in residential out-of-home care history fulfill the criteria for any mental disorder, with similar rates in adulthood and indifferent across child welfare and juvenile justice measures. Based on the similarly large stability of psychopathology from adolescence into adulthood in both samples, our results further support the approach of a shared placement in residential care institutions based on their treatment needs [72]. It is particularly important to provide early identification, continuity in cooperation between child and adolescent psychiatric services and the placing juvenile justice and child welfare agencies, and well-prepared transitions [15, 74, 76] for young people with mental health issues (i.e., liaison work; [34]). To sum up, future cross-national studies with larger samples should focus on individual factors and trajectories influencing psychopathology into adulthood across residential care settings and jurisdictions.

## Data Availability

The data that support the findings of this study can be requested from the first author and will be made available by the author.

## Supplementary material

For supplementary material accompanying this paper visit https://doi.org/10.1192/j.eurpsy.2022.30. click here to view supplementary material
