# Mental disorders into adulthood among adolescents placed in residential care: A prospective 10-year follow-up study – CORRIGENDUM

**DOI:** 10.1192/j.eurpsy.2025.22

**Published:** 2025-04-28

**Authors:** Süheyla Seker, Cyril Boonmann, Delfine d’Huart, David Bürgin, Klaus Schmeck, Nils Jenkel, Martin Steppan, Alexander Grob, Hilma Forsman, Jörg M. Fegert, Marc Schmid

**Keywords:** Developmental psychopathology, longitudinal study, mental disorders, residential care, corrigendum

In the analysis of the published article, a variable from the Structured Clinical Interview for DSM-IV-TR Axis II Personality Disorders (SCID-II) assessing antisocial behaviors before age 15 (similar to parts of the conduct disorder criteria measured with the Kiddie Schedule for Affective Disorders and Schizophrenia—Present and Lifetime Version [K-SADS-PL]) was mistakenly included as an independent diagnosis within the antisocial behavior disorders syndrome. However, this variable constitutes a prerequisite and forms part of the diagnostic criteria for antisocial personality disorder itself and should not have been treated as a separate diagnosis. This misclassification was applied at both assessment points (adolescence and young adulthood), resulting in inflated prevalence rates for antisocial behavior disorders and, consequently, for externalizing disorders, and any mental disorder. The analyses were revised and the corrected syntax excluding the misclassified variable was applied. Since conduct disorder was assessed with the K-SADS-PL in adolescence, the correction led to slight changes in prevalence rates in adolescence and larger changes in adulthood. The main findings of the study and the significance of the overall stability of general psychopathology remain unaffected. The corrected materials are provided below.

1. In the “Results” paragraph of the Abstract section, the prevalence rates of any mental disorder were corrected:
  In the total sample, prevalence rates of 69% and 63% for any mental disorder were found in adolescence (child welfare: 65%; juvenile justice: 78%), and adulthood (child welfare: 62%; juvenile justice: 67%) respectively.
2. In the “Statistical analyses” paragraph of the Methods section, the correlations of the “externalizing antagonistic” and “externalizing inhibited” were corrected, respectively:
  Due to a high correlation of the “externalizing antagonistic” and “externalizing disinhibited” disorder spectra in adolescence (child welfare: r_tet_ = 0.99; juvenile justice: r_tet_ = 0.91) and in adulthood (child welfare: r_tet_ = 0.84; juvenile justice: r_tet_ = 0.80), both spectra were grouped together in a single category labeled “externalizing disorders” spectrum.
3. In the “Prevalence rates of mental disorders” paragraph of the Results section, the prevalence rates of any mental disorder, antisocial behavior disorders, and externalizing disorders were corrected. Additionally, no significant differences were found for adult antisocial behavior disorders between child welfare and juvenile justice groups:
  In adolescence, 68.6% (95% CI = 57.7, 79.4) of the total sample showed any mental disorder (see Table 2), and in adulthood 62.9% (95% CI = 51.5, 74.2) of the participants had any mental disorder (see Table 3). Externalizing disorders (*n* = 41, 58.6% [95% CI = 47.0, 70.1) and internalizing disorders (*n* = 20, 28.6% [95% CI = 18.0, 39.2) were the most prevalent disorder spectra in adolescence, which was in line with the pattern in adulthood (externalizing disorders: *n* = 40, 57.1% [95% CI = 45.5, 68.7]; internalizing disorders: *n* = 26, 37.1% [95% CI = 25.8, 48.7]). For specific disorder groups, the highest prevalence rate in adolescence was found for antisocial behavior disorders (*n =* 37, 52.9% [95% CI = 41.2, 64.6]), followed by distress disorders (*n* = 16, 22.9% [95% CI = 13.0, 32.7]), substance abuse (*n* =15, 21.4% [95% CI = 11.8, 31.0]), and fear disorders (*n* = 9, 12.9% [95% CI = 5.0, 20.7]). In adulthood, the highest prevalence was found for substance abuse (*n* = 29, 41.4% [95% CI = 29.9, 53.0]), followed by antisocial behavior disorders (*n* = 24, 34.3% [95% CI = 23.2, 45.4]), distress disorders (*n* = 23, 32.9% [95% CI = 21.9, 43.9]) and fear disorders (*n* = 11, 15.7% [95% CI = 7.2, 24.2]). In adolescence, juvenile justice placed youths showed substance abuse more often compared to child welfare placed adolescents (χ^2^(1) = 3.10, *p* < .05).

**Table 2.** Prevalence Rates and Univariate Group Differences of Adolescent Mental Disorders in Child Welfare and Juvenile Justice Samples (% [n])

Disorder group	Adolescent mental disorders						Test statistic
	Total (*N* = 70)	95% CI	Child welfare (*n* = 52)	95% CI	Juvenile justice (*n* = 18)	95% CI	
Fear disorders	12.9 (9)	5.0, 20.7	11.5 (6)	2.9, 20.2	16.7 (3)	0, 33.9	χ^2^(1) = 0.02, n.s.
Distress disorders	22.9 (16)	13.0, 32.7	21.2 (11)	10.1, 32.3	27.8 (5)	7.1, 48.5	χ^2^(1) = 0.06, n.s.
Mania disorders	1.4 (1)	0, 4.2	1.9 (1)	0, 5.7	0 (0)	N/A	χ^2^(1) = 0.00, n.s.
Eating disorders	0 (0)	N/A	0 (0)	N/A	0 (0)	N/A	N/A
Substance abuse	21.4 (15)	11.8, 31.0	15.4 (8)	5.6, 25.2	38.9 (7)	16.4, 61.4	χ^2^(1) = 3.10^*^
Antisocial behavior disorders	52.9 (37)	41.2, 64.6	50.0 (26)	36.4, 63.6	61.1 (11)	38.6, 83.6	χ^2^(1) = 0.92, n.s.
Disorder spectrum							
Thought disorders	4.3 (3)	0, 9.0	1.9 (1)	0, 5.7	11.1 (2)	0, 25.6	χ^2^(1) = 0.97, n.s.
Internalizing disorders	28.6 (20)	18.0, 39.2	26.9 (14)	14.9, 39.0	33.3 (6)	11.6, 55.1	χ^2^(1) = 0.93, n.s.
Externalizing disorders	58.6 (41)	47.0, 70.1	53.9 (28)	40.3, 67.4	72.2 (13)	51.5, 92.9	χ^2^(1) = 1.81, n.s.
Detachment disorders	5.7 (4)	0.3, 11.2	3.9 (2)	0, 9.1	11.1 (2)	0, 25.6	χ^2^(1) = 0.31, n.s.
Any mental disorder	68.6 (48)	57.7, 79.4	65.4 (34)	52.5, 78.3	77.8 (14)	57.7, 79.4	χ^2^(1) = 0.47, n.s.

*Note.* CI = Confidence interval. n.s. = Not significant. N/A = Not applicable.

* *p* < .05

**Table 3.** Prevalence Rates and Univariate Group Differences of Adult Mental Disorders in Child Welfare and Juvenile Justice Samples (% [n])

Disorder group	Adult mental disorders						Test statistic
	Total (*N* = 70*)*	95% CI	Child welfare (*n* = 52)	95% CI	Juvenile justice (*n* = 18)	95% CI	
Fear disorders	15.7 (11)	7.2, 24.2	17.3 (9)	7.0, 27.6	11.1 (2)	0, 25.6	χ^2^(1) = 0.06, n.s.
Distress disorders	32.9 (23)	21.9, 43.9	30.8 (16)	18.2, 43.3	38.9 (7)	16.4, 61.4	χ^2^(1) = 0.12, n.s.
Mania disorders	1.4 (1)	0, 4.2	1.9 (1)	0, 5.7	0 (0)	N/A	χ^2^(1) = 0.00, n.s.
Eating disorders	N/A	N/A	N/A	N/A	N/A	N/A	N/A
Substance abuse	41.4 (29)	29.9, 53.0	42.3 (22)	28.9, 55.7	38.9 (7)	16.4, 61.4	χ^2^(1) = 0.00, n.s.
Antisocial behavior disorders	34.3 (24)	23.2, 45.4	32.7 (17)	36.4, 63.6	38.9 (7)	16.4, 61.4	χ^2^(1) = 0.04, n.s
Disorder spectrum							
Thought disorders	10.0 (7)	3.0, 17.0	5.8 (3)	0, 12.1	22.2 (4)	3.0, 41.4	χ^2^(1) = 2.40, n.s.
Internalizing disorders	37.1 (26)	25.8, 48.5	36.5 (19)	23.5, 49.6	38.9 (7)	11.6, 55.1	χ^2^(1) = 0.00, n.s.
Externalizing disorders	57.1 (40)	45.5, 68.7	53.9 (28)	40.3, 67.4	66.7 (12)	44.9, 88.4	χ^2^(1) = 0.45, n.s
Detachment disorders	10.0 (7)	3.0, 17.0	11.5 (6)	2.9, 20.2	5.6 (1)	0, 16.1	χ^2^(1) = 0.08, n.s.
Any mental disorder	62.9 (44)	51.5, 74.2	61.5 (32)	48.3, 74.8	66.7 (12)	47.1, 90.0	χ^2^(1) = 0.01, n.s

*Note.* CI = Confidence interval. n.s. = Not significant. N/A = Not applicable. Eating disorders are not included as a diagnosis in the *Structured Clinical Interview for DSM5 Disorders* (SCID-5) and were thus not assessed in adulthood.
4. The prevalence rates of any mental disorder, antisocial behavior disorders, and externalizing disorders were corrected in Table 2 and Table 3.

1. In the “Trajectories of adolescent and adult mental disorders” paragraph of the Results section, the distribution of participants across the trajectory groups were corrected:
  First, the trajectory group with any mental disorder at baseline and follow-up was the largest group (*n* = 35, 50.0%) in the total sample, followed by the trajectory group with a mental disorder that had improved through adulthood (*n* = 13, 18.6%), the group without a mental disorder at both baseline and follow-up (*n* = 13, 18.6%), and, finally, the group with a mental disorder at follow-up but not at baseline (*n* = 9, 12.9%).
2. The prevalence rates of the group differences between mental disorder trajectory groups with sociodemographic characteristics in the Table 3 of the Supplementary Material was corrected.

1. The estimates in the bivariate correlation matrices (Figures 2-4) in the Supplementary Material were corrected:

1. In the “Trajectories of adolescent and adult mental disorders” paragraph of the Results section, the model estimates for the confirmatory factor analysis were corrected (updated text and Figure 1). Additionally, the factor loading of externalizing disorders was significant for the adult general psychopathology latent variable. Finally, the estimates of the multi-group measurement invariance analysis were updated (text and Figure 2):
  Third, the two-factor CFA showed a nonsignificant χ^2^-test (χ^2^(15) = 13.57, *p* = .56), indicating a good model fit. Furthermore, the CFA also provided good fit indices for RMSEA = 0.00, CFI = 1.00, and TLI = 1.01.
  For the adult general psychopathology latent variable, all factor loadings of the mental disorder groups were significant (all *p* < .01).
  There was a significant covariance between adolescent and adult general psychopathology (*b* = 0.76, SE = 0.13, *p* < .001), indicating temporal stability of general psychopathology.
  A measurement invariance analysis comparing adolescent and adult mental disorders between the child welfare and juvenile justice groups revealed that the scalar invariance was the best suited model (χ^2^(6) = 4.73).
  Lasty, Fisher’s z test for the adolescent general psychopathology and adult general psychopathology factor scores revealed no significant differences between the child welfare and juvenile justice samples (*z* = –1.09, *p* = .28), indicating a similarly large temporal stability of general psychopathology in both samples (see Figure 2).

**Figure 1.**
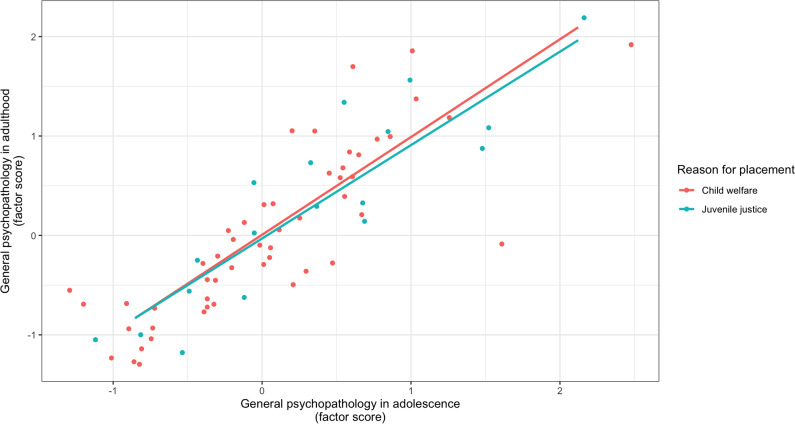
Two-Factor Confirmatory Factor Analysis of Adolescent and Adult Mental Disorders in the Total Sample (n = 70). *Note*. CI = Confidence interval. The 95% confidence interval for factor loadings is presented in brackets. **p < .01, ***p < .001

**Figure 2.**
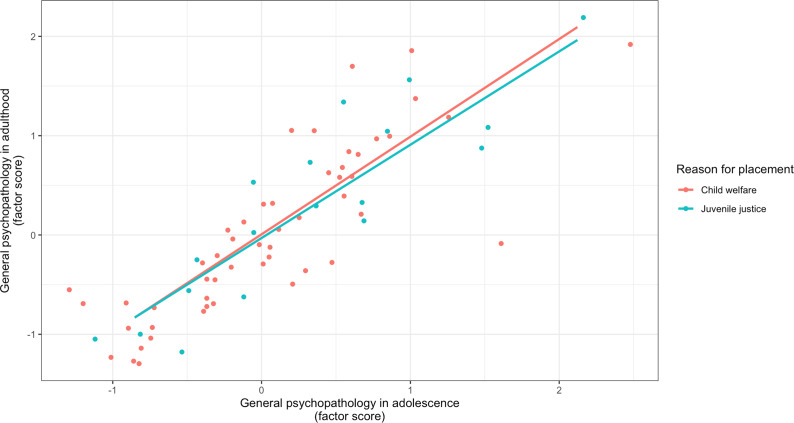
Temporal Stability of Adolescent and Adult General Psychopathology in the Child Welfare (n = 52) and Juvenile Justice Sample (n = 18). *Note.* The x- and y- axes are scaled according to the factor score of adolescent and adult general psychopathology derived from the multi-group confirmatory factor analysis. The factor scores between both groups did nod differ significantly between the child welfare and juvenile justice group.
2. The prevalence rates in the following sentences of the Discussion were corrected:
  Almost 69% of adolescents in out-of-home care showed any mental disorder. The rates for any mental disorder were similar in both the child welfare (65%) and the juvenile justice (78%) samples.
  In the present study, the prevalence rate of 63% for any mental disorder among the total sample in adulthood (child welfare: 62%; juvenile justice: 67%) is higher than in a recent meta-analysis of mental disorders in adults formerly placed in out-of-home care by child welfare or juvenile justice authorities [35], and also far higher than a pooled prevalence rate of 18% for any mental disorder in the general adult population [68].
  Within our total sample, around 50% of participants showed persistent general psychopathology.

## Supplementary Materials

**Table 3.** Prevalence Rates and Group Differences for Mental Disorder Trajectory Groups With Sociodemographic Characteristics

Characteristics	Without a mental disorder at baseline and follow-up (*n* = 13)	Mental disorder at follow-up but not at baseline (*n* = 9)	Mental disorder at baseline but not at follow-up (*n* = 13)	Mental disorder at baseline and follow-up (*n* = 35)	Test statistic
Gender (% [*n*])					χ^2^(3) = 3.29, n.s.
Female	15.4 (2)	44.4 (4)	46.2 (6)	37.1 (13)	
Male	84.6 (11)	55.6 (5)	53.9 (7)	62.9 (22)	
Age in years (*M*, *SD*)	14.8 (1.4)	16.3 (1.5)	15.8 (1.8)	15.7 (1.3)	*F*(3) = 2.2, n.s.
Swiss citizenship (% [*n*])	76.9 (10)	77.8 (7)	92.3 (12)	88.6 (31)	χ^2^(3) = 2.00, n.s.
Reason for placement (% [*n*])					χ^2^(3) = 1.79, n.s.
Child welfare (civil law)	76.9 (10)	88.9 (8)	76.9 (10)	68.6 (24)	
Juvenile justice (criminal law)	23.1 (3)	11.1 (1)	23.1 (3)	31.3 (11)	
Number of placements (*M*, *SD*)	2.5 (2.5)	3.4 (2.3)	4.0 (4.9)	3.9 (2.4)	*F*(3) = 0.76, n.s.
Age at first entry into care in years	7.6 (6.6)	11.4 (4.0)	10.1 (4.3)	10.9 (4.5)	*F*(3) = 0.50, n.s.
Duration of care in years	6.9 (5.9)	8.3 (5.5)	4.9 (2.9)	6.0 (4.6)	*F*(3) = 1.03, n.s.

*Note.* n.s. = Not significant. *M* = Mean. *SD* = Standard deviation. Raw numbers across cells do not add up to the total sample size due to missing data for some variables.

**Figure 2.**
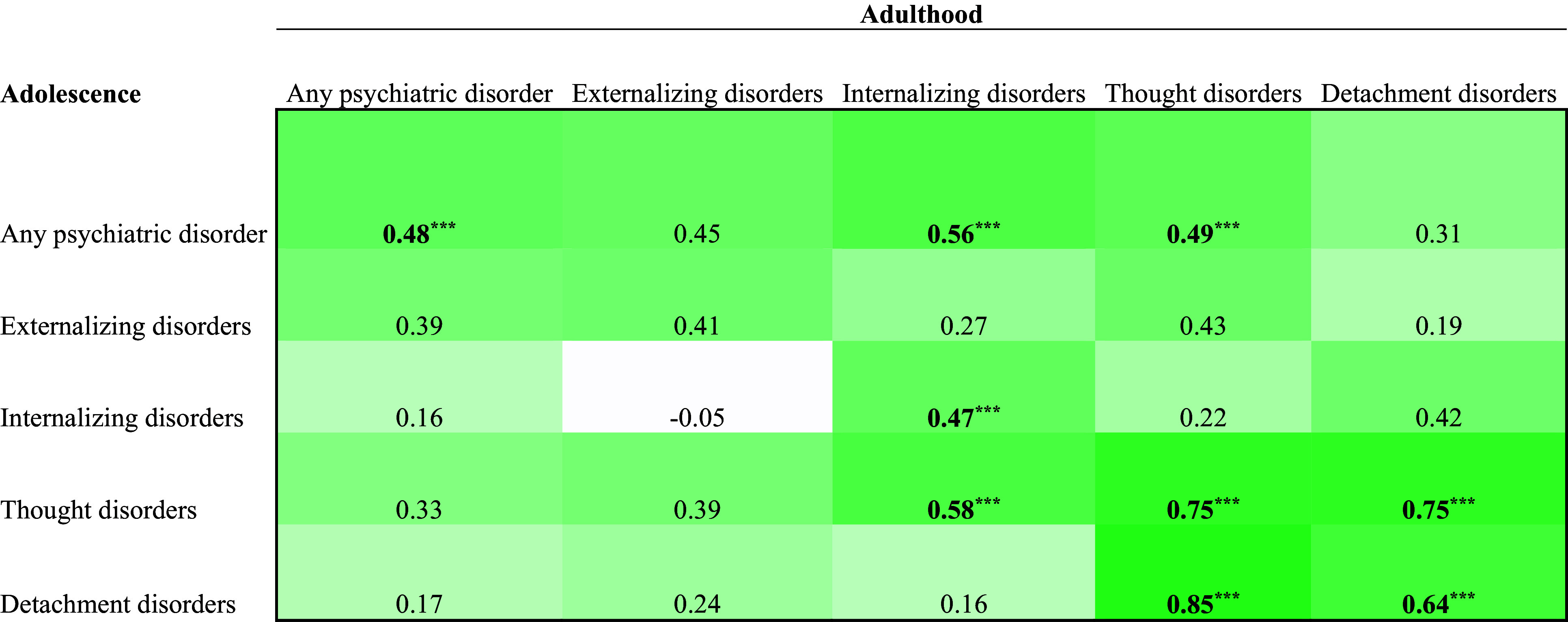
Correlation Matrix of Adolescent and Adult Mental Disorders in the Total Sample (n = 70). *Note*. Bolded values are significant at a Bonferroni corrected a level (.05/25 = .002). ***p < .001

**Figure 3.**
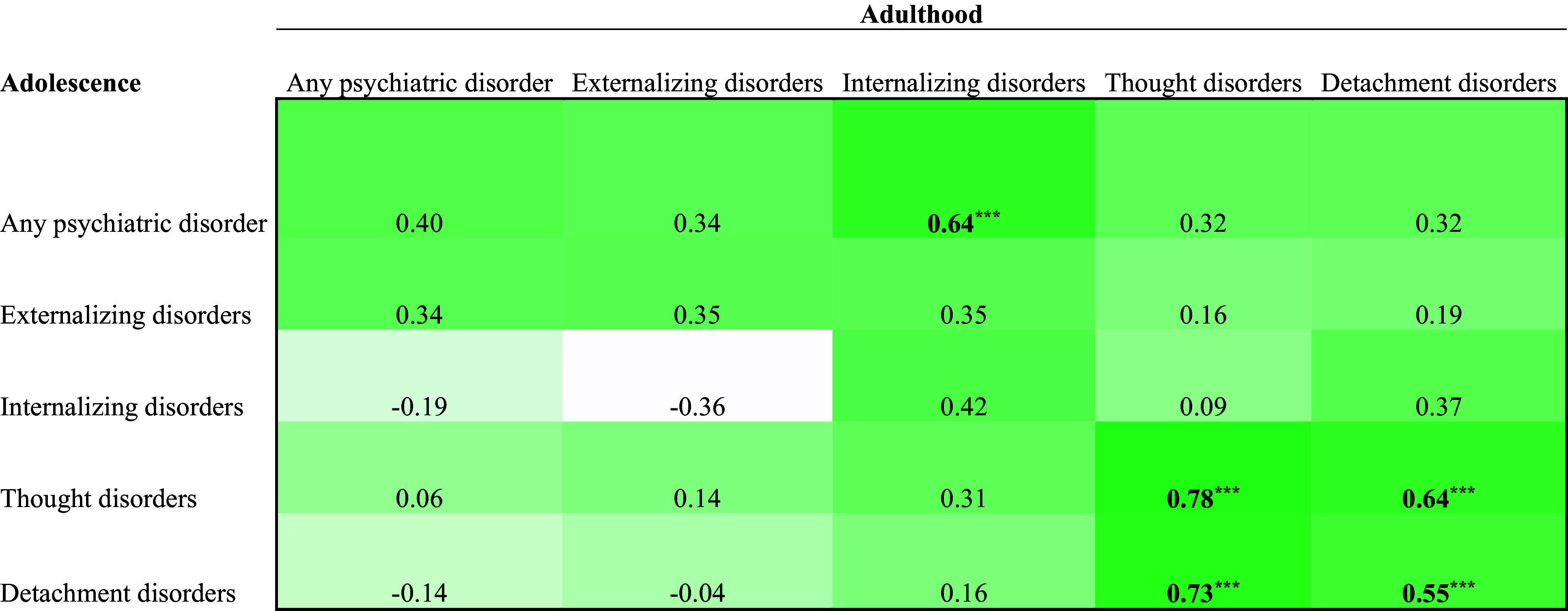
Correlation Matrix of Adolescent and Adult Mental Disorders in the Child Welfare Sample (n = 52). *Note*. Bolded values are significant at a Bonferroni corrected a level (.05/25 = .002). ***p < .001

**Figure 4.**
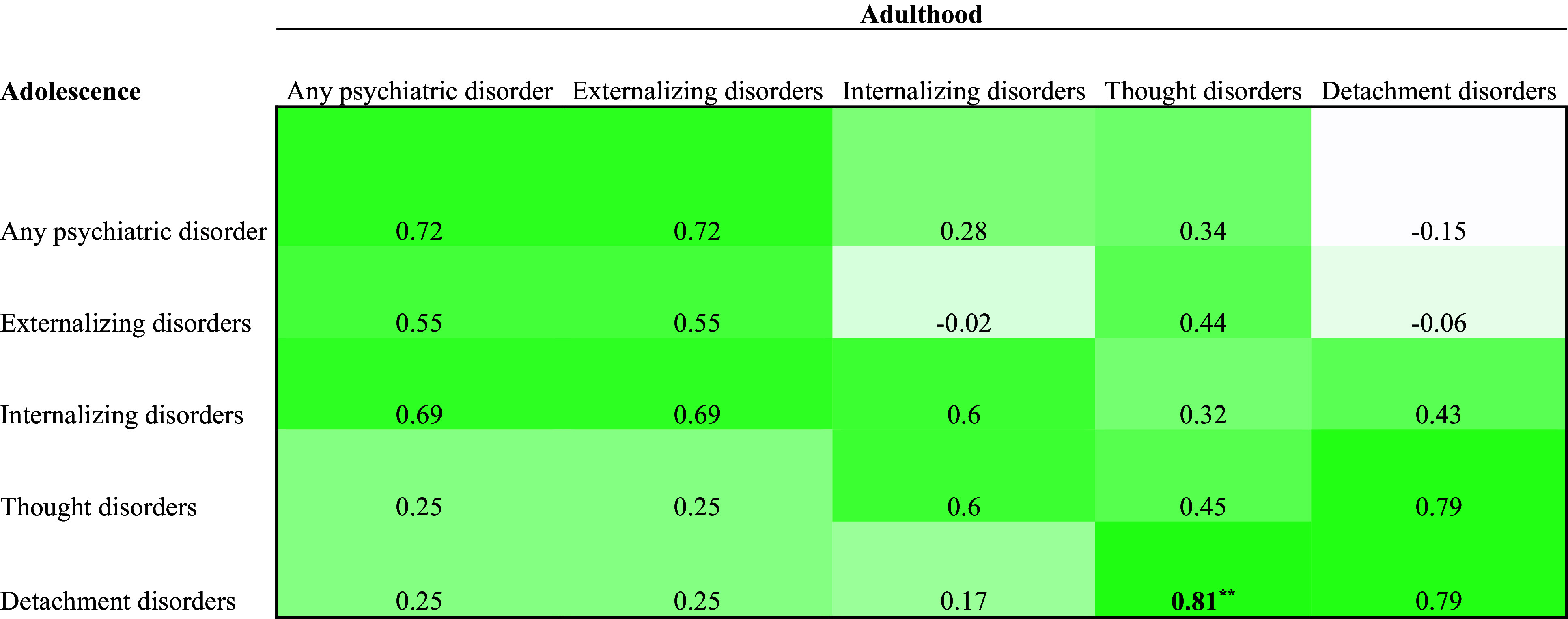
Correlation Matrix of Adolescent and Adult Mental Disorders in the Juvenile Justice Sample (n = 18). *Note*. Bolded values are significant at a Bonferroni corrected a level (.05/25 = .002). **p < .01
