# Supplementary material for: Mental disorders into adulthood among adolescents placed in residential care: A prospective 10-year follow-up study
Source: Eur Psychiatry. 2022 Jun 22;65(1):e40. doi: 10.1192/j.eurpsy.2022.30 (PMC9280920; doi:10.1192/j.eurpsy.2022.30)
Supplement: Supplementary file 1 [file S092493382200030Xsup001.docx]

**Supplementary Material**

**Mental Disorders Into Adulthood Among Adolescents Placed in Residential Care Into Adulthood: A Prospective 10-Year Follow-up Study**

**Content**

| Figure 1. | Flowchart of Study Sample |
| --- | --- |
| Table 1. | Sample Attrition Analysis |
| Table 2. | Grouping of Mental Disorders According to the HiTOP model |
| Table 3. | Prevalence Rates and Group Differences for Mental Disorder Trajectory Groups With Sociodemographic Characteristics |
| Figure 2. | Correlation Matrix of Adolescent and Adult Mental Disorders in the Total Sample (*n* = 70) |
| Figure 3. | Correlation Matrix of Adolescent and Adult Mental Disorders in the Child Welfare Sample (*n* = 52) |
| Figure 4. | Correlation Matrix of Adolescent and Adult Mental Disorders in the Juvenile Justice Sample (*n* = 18) |

Figure 1

*Flowchart of Study Sample*

Included in the baseline study

(*N* = 592)

Reason for drop-out (*n* = 249):

- Did not provide informed consent

(*n* = 81)

- > 18 years at baseline (*n* = 68)
- Voluntary placements (*n* = 100)

**Baseline (MAZ.)**

Eligible follow-up participants (*N* = 343)

Reason for drop-out (*n* = 112):

- Declined to participate (*n* = 39)
- Did not provide informed consent

(*n* = 13)

- Could not be reached (*n* = 52)
- Deceased (*n* = 8)

Included in the follow-up study
(*N* = 231)

**Follow-Up (JAEL)**

Reason for drop-out (*n* = 161):

- Missing data on different variables

(n = 161)

Included in the final analyses

(*N* = 70)

| Table 1  *Sample Attrition Analysis* | | | |
| --- | --- | --- | --- |
| Characteristics | Included study participants (*N* = 70) | Non-included participants (*N* = 273) | Test statistic |
| Gender (% [*n*]) |  |  | χ^2^(1) = 0.53, n.s. |
| Female | 35.7 (25) | 41.4 (113) |  |
| Male | 64.5 (45) | 58.6 (160) |  |
| Age in years (*M*, *SD*) | 15.65 (1.48) | 14.84 (2.53) | *t*(185.22) = –3.49, n.s. |
| Swiss citizenship (% [*n*]) | 85.7 (60) | 83.5 (228) | χ^2^(1) = 0.07, n.s. |
| Reason for placement (% [*n*]) |  |  |  |
| Child welfare (civil law) | 74.3 (52) | 81.0 (221) |  |
| Juvenile justice (criminal law) | 25.7 (18) | 19.1 (52) |  |
| Number of placements (*M*, *SD*) | 3.63 (3.02) | 4.24 (4.14) | *t*(75.99) = 0.86, n.s. |
| Age at first entry into care in years (*M*, *SD*) | 10.23 (4.68) | 10.73 (5.57) | *t*(33.38) = 0.45, n.s. |
| Duration of care in years (*M*, *SD*) | 6.28 (4.75) | 6.71 (3.96) | *t*(96.31) = 0.51, n.s. |
| Adolescent externalizing disorders (% [*n*]) | 64.3 (45) | 62.3 (114) | χ^2^(1) = 0.02, n.s. |
| Adolescent internalizing disorders (% [*n*]) | 28.6 (20) | 36.1 (66) | χ^2^(1) = 0.96, n.s. |
| Adolescent any mental disorder (% [*n*]) | 72.9 (51) | 74.5 (136) | χ^2^(1) = 0.01, n.s. |
| *Note. M* = Mean. *SD* = Standard deviation. n.s. = Not significant. The number of participants does not add up to *N* = 273 273 for non-included participants in the adolescent externalizing, internalizing, and any mental disorder variables due to missing data. | | | |

| Table 2  *Grouping of Mental Disorders According to the HiTOP model* | |
| --- | --- |
|  | Grouping of disorders according to ICD-10 |
| Disorder group |  |
| Fear disorders | F40–F42, F93 |
| Distress disorders | F32–F34, F38–F38, F41, F43, F51, F93.3, F93.8, F60.31 |
| Mania disorders | F30–F31, F32.3 |
| Eating disorders | F50.1, F50.3 |
| Substance abuse | F10–F25 |
| Antisocial behavior disorders | F60.2, F60.3, F60.4, F60.81, F60.0 F63.2, F90–F92, F94 |
| Disorder spectrum |  |
| Thought disorders | F60.0, F60.1, F32.3 |
| Internalizing disorders | F32–F34, F38–F38, F41, F43, F40–F42, F50.1, F50.3, F51, F60.31, F93.3, F93.8, F93 |
| Externalizing disorders | F10–F25, F60.2, F60.3, F60.4, F60.81, F60.0, F63.2, F90–F92, F94 |
| Detachment disorders | F60.6, F60.7, F60.4, F60.1 |
| Any mental disorder | F10–F25, F32–F34, F38–F38, F41, F43, F40–F42, F50.1, F50.3, F51, F60.31, F93.3, F93.8, F93, F60.2, F60.3, F60.4, F60.81, F60.0, F60.1, F60.4, F60.6, F60.7, F63.2, F90–F92, F94 |
| *Note.* ICD-10 = *International Classification of Diseases 10th Revision.* Eating disorders are not included as a diagnosis in the Structured Clinical Interview for DSM5 Disorders (SCID-5) and were thus not assessed in adulthood. | |

| Table 3  *Prevalence Rates and Group Differences for Mental Disorder Trajectory Groups With Sociodemographic Characteristics* | | | | | |
| --- | --- | --- | --- | --- | --- |
| Characteristics | Without a mental disorder at baseline and follow-up (*n* = 4) | Mental disorder at follow-up but not at baseline (*n* = 15) | Mental disorder at baseline but not at follow-up (*n* = 6) | Mental disorder at baseline and follow-up (*n* = 45) | Test statistic |
| Gender (% [*n*]) |  |  |  |  | χ^2^(3) = 2.88, n.s. |
| Female | 0 (0) | 40.0 (6) | 50.0 (3) | 35.6 (16) |  |
| Male | 100.0 (4) | 60.0 (9) | 50.0 (3) | 64.4 (29) |  |
| Age in years (*M*, *SD*) | 14.23 (2.33) | 15.72 (1.37) | 15.86 (2.05) | 15.73 (1.33) | *F*(3) = 1.36, n.s. |
| Swiss citizenship (% [*n*]) | 100.0 (4) | 73.3 (11) | 83.3 (5) | 88.9 (40) | χ^2^(3) = 2.94, n.s. |
| Reason for placement (% [*n*]) |  |  |  |  | χ^2^(3) = 4.65, n.s. |
| Child welfare (civil law) | 75.2 (3) | 86.7 (13) | 100.0 (6) | 85.7 (30) |  |
| Juvenile justice (criminal law) | 25.0 (1) | 13.3 (2) | 0 (0) | 33.3 (15) |  |
| Number of placements (*M*, *SD*) | 4.00 (4.24) | 2.87 (1.99) | 5.50 (7.29) | 3.60 (2.26) | *F*(3) = 1.11, n.s. |
| Age at first entry into care in years | 7.54 (4.64) | 7.73 (7.01) | 6.88 (3.09) | 11.53 (4.22) | *F*(3) = 1.17, n.s. |
| Duration of care in years | 9.75 (5.25) | 7.20 (5.99) | 5.17 (3.19) | 5.80 (4.37) | *F*(3) = 1.28, n.s. |
| *Note.* n.s. = Not significant. *M* = Mean. *SD* = Standard deviation. Raw numbers across cells do not add up to the total sample size due to missing data for some variables. | | | | | |

Figure 2

*Correlation Matrix of Adolescent and Adult Mental Disorders in the Total Sample (n = 70)*

|  | **Adulthood** | | | | |
| --- | --- | --- | --- | --- | --- |
| **Adolescence** | Any psychiatric disorder | Externalizing disorders | Internalizing disorders | Thought disorders | Detachment disorders |
| Any psychiatric disorder | 0.23 | 0.27 | **0.48^***^** | 0.44^**^ | 0.25 |
| Externalizing disorders | 0.23 | 0.38^*^ | 0.13 | 0.36^*^ | 0.11 |
| Internalizing disorders | –0.03 | –0.09 | **0.47^**^** | 0.22 | 0.42** |
| Thought disorders | 0.01 | 0.06 | **0.58^***^** | **0.75^***^** | **0.75^***^** |
| Detachment disorders | –0.20 | –0.14 | 0.16 | **0.85^***^** | **0.64^***^** |

*Note.* Bolded values are significant at a Bonferroni corrected α level (.05/25 = .002).

^*^*p* < .05*,* ^**^*p* < .01, ^***^*p* < .001

Figure 3

*Correlation Matrix of Adolescent and Adult Mental Disorders in the Child Welfare Sample (n = 52)*

|  | **Adulthood** | | | | |
| --- | --- | --- | --- | --- | --- |
| **Adolescence** | Any psychiatric disorder | Externalizing disorders | Internalizing disorders | Thought disorders | Detachment disorders |
| Any psychiatric disorder | 0.05 | 0.11 | **0.57^***^** | 0.27 | 0.26 |
| Externalizing disorders | 0.07 | 0.26 | 0.22 | 0.09 | 0.11 |
| Internalizing disorders | –0.13 | –0.2 | 0.42 | 0.09 | 0.37 |
| Thought disorders | –0.21 | –0.15 | 0.31 | **0.78^***^** | **0.64^***^** |
| Detachment disorders | –0.43* | –0.37 | 0.16 | **0.73^***^** | **0.55^***^** |

*Note.* Bolded values are significant at a Bonferroni corrected α level (.05/25 = .002).

^*^*p* < .05*,* ^**^*p* < .01, ^***^*p* < .001

Figure 4

*Correlation Matrix of Adolescent and Adult Mental Disorders in the Juvenile Justice Sample (n = 18)*

|  | **Adulthood** | | | | |
| --- | --- | --- | --- | --- | --- |
| **Adolescence** | Any psychiatric disorder | Externalizing disorders | Internalizing disorders | Thought disorders | Detachment disorders |
| Any psychiatric disorder | 0.67 | 0.67 | 0.10 | 0.23 | –0.24 |
| Externalizing disorders | 0.58 | 0.58 | –0.21 | 0.34 | –0.15 |
| Internalizing disorders | 0.02 | 0.02 | 0.60 | 0.32 | 0.43 |
| Thought disorders | –0.37 | –0.37 | 0.60 | 0.45 | 0.79^*^ |
| Detachment disorders | –0.37 | –0.37 | 0.17 | **0.81^**^** | 0.79^**^ |

*Note.* Bolded values are significant at a Bonferroni corrected α level (.05/25 = .002).

^*^*p* < .05*,* ^**^*p* < .01
